# Prospective role of lusianthridin in attenuating cadmium-induced functional and cellular damage in rat thyroid

**DOI:** 10.1016/j.heliyon.2024.e27080

**Published:** 2024-02-27

**Authors:** Teng Gao, Sijia Luo, Hongguang Li, Zijie Su, Qinghui Wen

**Affiliations:** a Department of Thyroid Surgery, Henan Provincial People’s Hospital, Zhengzhou University People’s Hospital, Zhengzhou, Henan, 450003, China; b Department of General Surgery, General Hospital of Central Theater Command, Wuhan, Hubei, 430070, China; c Department of Clinical Laboratory, Dongguan People's Hospital, Dongguan, Guangdong, 523059, China

**Keywords:** Lusianthridin, Deiodinase, Lipid, Antioxidant, mRNA

## Abstract

The thyroid represents the most prevalent form of head and neck and endocrine cancer. The present investigation demonstrates the anticancer effects of Lusianthridin against cadmium (Cd)-induced thyroid cancer in rats. Swiss Wistar rats were utilized in this experimental study. Cd was employed to induce thyroid cancer, and the rats were divided into different groups, receiving oral administration of Lusianthridin (20 mg/kg) for 14 days. Thyroid parameters, deiodinase levels, hepatic parameters, lipid parameters, and antioxidant parameters were respectively estimated. The mRNA expression was assessed using real-time reverse transcriptase polymerase chain reaction (RT-PCR). Lusianthridin significantly (P < 0.001) improved protein levels, T4, T3, free iodine in urine, and suppressed the level of TSH. Lusianthridin significantly (P < 0.001) enhanced the levels of FT3, FT4, and decreased the level of rT3. Lusianthridin significantly (P < 0.001) reduced the levels of D1, D2, D3, and enhanced the levels of hepatic parameters like AST, ALT. Lusianthridin remarkably (P < 0.001) altered the levels of lipid parameters such as LDL, total cholesterol, HDL, and triglycerides; antioxidant parameters viz., MDA, GSH, CAT, and SOD. Lusianthridin significantly altered the mRNA expression of Bcl-2, Bax, MEK1, ERK1, ERK2, *p*-eIf2α, GRP78, eIf2α, and GRP94. The results clearly state that Lusianthridin exhibits protective effects against thyroid cancer.

## 1 Introduction

Thyroid cancer is the most prevalent form of endocrine and head-and-neck cancer [1]. Thyroid cancer refers to the abnormal and uncontrolled growth of cells in the thyroid gland. The incidence of thyroid cancer remained stable until the 1990s, after which it increased dramatically [2]. The report suggests that thyroid cancer accounts for 2.1% of all cancer diagnoses worldwide. It also indicates that the majority of patients affected by thyroid cancer are women, with approximately more than 77% of thyroid cancer cases being diagnosed among women [3,4]. Overall, the incidence of thyroid cancer increased from 4.9 per 100,000 population to 14.7 per 100,000 population in 2011 [5]. As per the Global Cancer Statistics 2018, approximately 41,000 new cases of thyroid cancer were identified in 2018, and thyroid cancer ranked 6th among all types of cancer mortality [4]. Environmental variables, including contaminants such as bisphenol A, organochlorine chemicals, triclosan, polychlorinated biphenyls, thiocyanates, heavy metals, nitrates, and polybrominated diphenyl ethers, increase the risk of autoimmune diseases, thyroid dysfunction, and thyroid cancer.

Cd is increasingly observed in the biological system and the environment, among all the heavy metal pollutants [6]. The Food and Agriculture Organization (FAO) and the World Health Organization (WHO) both acknowledge that Cd is highly detrimental to human health [7,8]. However, with the acceleration of industrialization, economic growth, and agricultural modernization, Cd contamination and its effects on ecosystems are increasingly recognized as a severe worldwide danger [8,9]. As per the report, approximately more than 22,000 tonnes of Cd compounds have been deposited in the environment, primarily in the soil. Cd is a metal that can accumulate in soil and plants, particularly in areas with high levels of environmental pollution [8,10]. Plants growing in contaminated soil can absorb Cd through their roots and accumulate it in their tissues, including edible parts such as leaves, fruits, and grains. Studies have shown that when the Cd concentration in the soil reaches 11 mg/kg, the Cd concentration in rice can reach levels as high as 1.19 mg/kg [11]. This gradual accumulation of Cd in the food chain can significantly amplify its toxicity, as animals and humans who consume these contaminated crops can also accumulate Cd in their bodies over time [5,11]. Heavy metals like Cd may seriously impair the endocrine system's ability to function. Cd exposure can lead to a range of health issues, including kidney damage, lung damage, and an elevated risk of cancer, particularly thyroid cancer. Cd is typically found in cigarette smoke, phosphate fertilizer, food, nickel-cadmium batteries, and mines [12,13]. Cd enters the body via the alveolar epithelium and gastrointestinal tract, passes through systemic circulation, and finally binds with albumin. Cd can enter the body through the gastrointestinal tract by ingesting contaminated food and water, or through the inhalation of contaminated air, which can deposit in the lungs and be absorbed through the alveolar epithelium [14,15]. Once Cd enters the body, it is transported through the bloodstream, primarily bound to the protein albumin. The liver, one of the major organs, metabolizes and detoxifies Cd. Additionally, in response to Cd exposure, the liver produces a protein called metallothionein (MT) [11]. Metallothionein is a small, cysteine-rich protein that can bind to heavy metals like Cd and aid in their detoxification. It can be expressed when exposed to various environmental stresses, such as heavy metals, and is present in various body tissues, including the liver, kidneys, and lungs. An essential defense mechanism against the detrimental effects of Cd is the induction of metallothionein production, which helps in the sequestration and detoxification of this poisonous metal in the body [5,11]. However, chronic exposure to Cd can overwhelm this protective mechanism, leading to the accumulation of Cd in the body and the development of toxicity over time [16,17].

Chronic exposure to Cd can harm the thyroid gland and interfere with thyroid hormone production [18]. Research indicates that thyroid dysfunction is indicated by a rise in serum thyrotropin (TSH) content in both rats and humans exposed to Cd. Additionally, studies have shown that Cd and selenium (Se) may interact in the thyroid gland [11]. Se is a necessary component of the deiodinases, enzymes that convert thyroid hormone from its inactive to active form. Cd can combine with Se to form insoluble complexes. This interaction can make it challenging for the thyroid gland to produce and utilize selenoproteins [19,20]. Cd exposure has also been linked to an increased risk of testicular autoimmunity and other autoimmune disorders, in addition to impairing thyroid function. One investigation assessed the levels of three heavy metals in the blood (Cd, Hg, and Pb) as well as the blood mRNA expression of 98 genes linked to stress, toxicity, inflammation, and autoimmune disease in 24 people [21,22]. The research discovered a link between increased gene expression for stress, toxicity, inflammation, and autoimmune responses and exposure to Cd [21]. Additionally, a study on more than 5000 Chinese adults discovered that thyroid hypofunction and serum thyroid autoantibody levels were directly associated with blood levels of both Cd and lead (Pb) [23]. Blood Cd exposure increased the likelihood of hypothyroidism gradually in males but not in women. Blood levels of Cd were positively correlated with women's tertiles of thyroglobulin autoantibodies (TgAb) and the hypothyroid state [5,23].

## 2 Material and method

### 2.1 Experimental protocol

In the current experimental work, male Swiss Wistar rats (150–200 g, 12–14 weeks old) were used. The rats were provided with standard feed and unlimited water. They were housed under typical laboratory conditions, with a temperature of 20 °C, 65% relative humidity, and a 12/12-h cycle of darkness and light. To acclimate to the environment, the rats were kept in the laboratory conditions for a week. The entire experimental study was conducted in accordance with the Guide for Care and Use of Laboratory Animals provided by the Institutional Animal Ethical Committee. The Institutional Animal Ethical Committee approved the entire protocol (2020-0106).

### 2.2 Experimental design

The rats were divided into different groups as follow.

Group A: normal group received the oral treatment of saline, Group B: normal + Lusianthridin (20 mg/kg)

Group C: treated with the cadmium.

Group D: treated with cadmium + Lusianthridin (20 mg/kg), respectively.

Lusianthridin was administered orally to the rats for a total of 14 days. Throughout the experimental investigation, measurements of body weight, food intake, and water consumption were recorded at regular intervals. After the 14-day treatment period, diethyl ether was used to induce unconsciousness in the rats, and blood was drawn by puncturing the retroorbital plexus. Ketamine and xylene were then utilized to euthanize the rats, and the thyroid tissue was successfully removed. The thyroid tissues were centrifuged, preserved in 10% natural buffered formalin, and fixed in glutaraldehyde (2.5%).

### 2.3 Thyroid hormones

The thyroid hormones such as T3 (SRP0114), thyroid stimulating hormone (TSH) (SE120135) and T4 (RAB0458) were analyzed using the colorimetric ELISA kit following the manufacture instruction (Sigma Aldrich, USA).

### 2.4 Hepatic parameters

ELISA kits were used for the estimation of hepatic parameters like alanine aminotransferase (ALT) (MAK052-1 KT) and aspartate aminotransferase (AST) (MAK055-1 KT) were estimated using the assay kit via following the manufacture instruction (Sigma Aldrich, USA).

### 2.5 Antioxidant parameters

The antioxidant parameters like malonaldehyde (MDA) (MAK085), superoxide dismutase (SOD) (19160), glutathione (GSH) (CS0260) and catalase (CAT) (MAK531) were estimated using the assay kit following the manufacture instruction (Sigma Aldrich, USA).

### 2.6 Deiodinase activity

The deiodinase such as D1, D2, and D3 were estimated in the liver homogenate via using the corresponding kits (Shanghai Jiang Lai Biotechnology Co., Ltd., Shanghai, China).

### 2.7 mRNA expression

Trizol reagent was used for the isolation to total RNA from the thyroid tissue. Subsequently, mRNA was reverse transcribed to cNDA using the oligonucleotide dT primers as per using the manufacture instructions. Platinum Taq polymerase was performed for RT-PCR analysis. The primer used in the current study was presented in Table 1.

**Table 1:** List of mRNA expression.

S. No	Gene	Primer	
		Forward	Reverse
**1**	Bax	AGGATGCGTCCACCAAGAA	CAAAGTAGAAGAGGGCAACCAC
**2**	Bcl-2	TGTGGCCTTCTTTGAGTTCG	TACCCAGCCTCCGTTATCCT
**3**	MEK1	TGACGCAGAAGCAGAAGGTG	TGAAGACCACTCCACCGTTG
**4**	ERK1	GGCTTTCTGACGGAGTATGTG	GGGGAACCCAAGATACCTAGA
**5**	ERK2	GGTTGTTCCCAAATGCTGAC	GCTCATCACTTGGGTCATAATACT
**6**	eIf2α	TAATCAATGTCGCTAACAAGGG	AAGTTGTAGGTTAGGCGTCCC
**7**	*p*-eIf2α	TTCTACAGAAACCATGCCCAT	TTGATAACTGCCATAGCCTGAT
**8**	GRP78	GCCAACTGTAACAATCAAGGTCT	TCAGGTGTCAGGCGGTTTT
**9**	GRP94	AGGTGTTGTGGATTCCGATGA	AGTTTAGCAAGCCGTGTTCG
**10**	β-actin	GTGCTATGTTGCTCTAGACTTCG	ATGCCACAGGATTCCATACC

### 2.8 Statistical analysis

The result of the study was presented as mean ± standard error means (SEM). All the treated groups were compared and analyzed using the Graphpad Prism version 8 (St. Louis, USA). Difference between the groups were considered as the significant with one way analysis of variance (ANOVA) using the Dunnett's test, where P < 0.05 was consider as significant.

## 3 Result

### 3.1 Body and organ weight

**Fig. 1:**
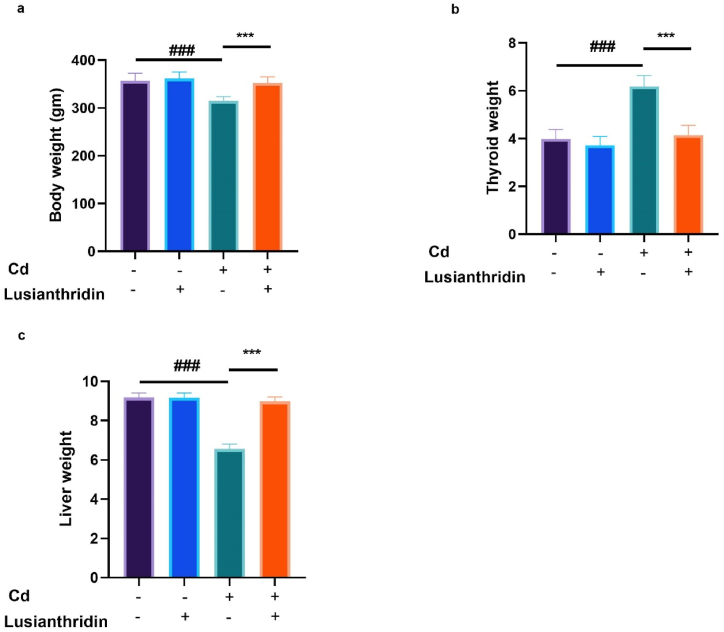
Showed the effect of Lusianthridin on the body and organ weight against the cadmium induced thyroid cancer. **a:** body weight, **b:** thyroid weight and **c:** liver weight. Group III compared with the normal control and ^**###**^P < 0.001was consider as significant. Group IV compared with the Group III and ***P < 0.001was consider as significant.

Cancer disease caused a decrease in both body and organ weight in the rats. Rats with thyroid cancer induced by Ca exhibited alteration in overall weight and organ weight, including the liver and thyroid tissue. However, thyroid cancer rats treated with lusianthridin showed a significant alteration (P < 0.001) in body weight (Fig. 1a), thymus weight (Fig. 1b) and hepatic weight (Fig. 1c).

### 3.2 Protein, T3, T4, TSH and free iodine

**Fig. 2:**
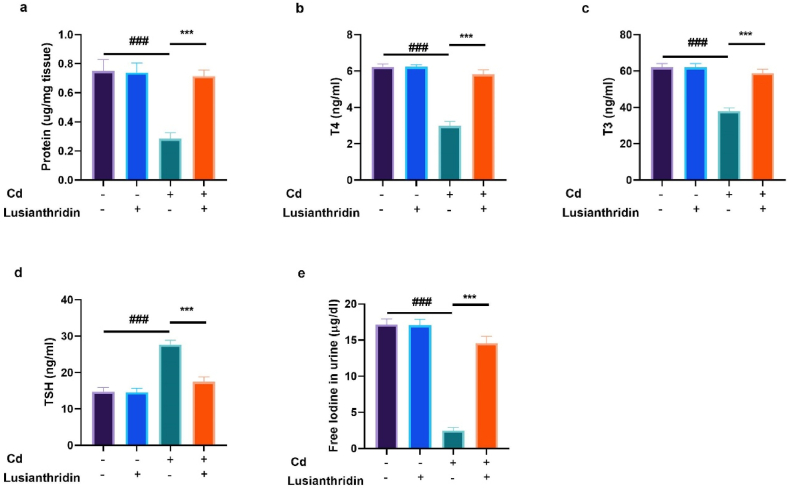
Showed the effect of Lusianthridin on the protein, T4, T3, TSH and free iodine in urine against the cadmium induced thyroid cancer. **a:** protein, **b:** T4, **c:** T3**, d:** TSH and **e:** free iodine in urine. Group III compared with the normal control and ^**###**^P < 0.001was consider as significant. Group IV compared with the Group III and ***P < 0.001was consider as significant.

During thyroid cancer, the levels of protein, T3, T4, TSH, and free iodine can be affected, depending on the type and stage of cancer. Rats in the Cd-induced thyroid cancer group exhibited altered levels of protein (Fig. 2a), T4 (Fig. 2b), T3 (Fig. 2c), TSH (Fig. 2d), and free iodine (Fig. 2e). Treatment with Lusianthridin remarkably restored the levels of protein, T3, T4, TSH, and free iodine.

### 3.3 FT3, FT4 and rT3

**Fig. 3:**
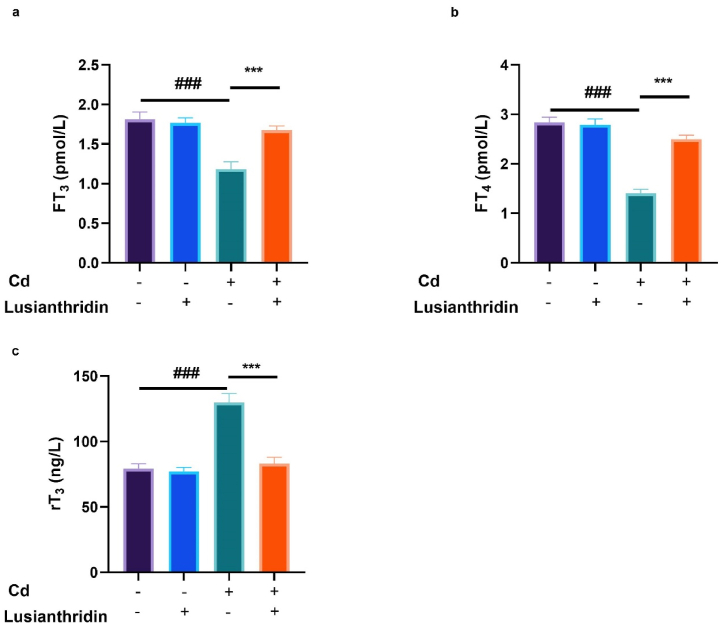
Showed the effect of Lusianthridin on the FT_3_, FT_4_ and rT_3_ against the cadmium induced thyroid cancer. **a:** FT_3_, **b:** FT_4_ and **c:** rT_3_. Group III compared with the normal control and ^**###**^P < 0.001was consider as significant. Group IV compared with the Group III and ***P < 0.001was consider as significant.

The levels of FT3, FT4, and rT3 can play a role in the diagnosis and management of thyroid cancer. Rats with Cd-induced thyroid cancer exhibited suppressed levels of FT3 (Fig. 3a) and FT4 (Fig. 3b), as well as an elevated level of rT3 (Fig. 3c). The group of rats treated with Lusianthridin exhibited alterations in the levels of FT3, FT4, and rT3.

### 3.4 Deiodinase level

**Fig. 4:**
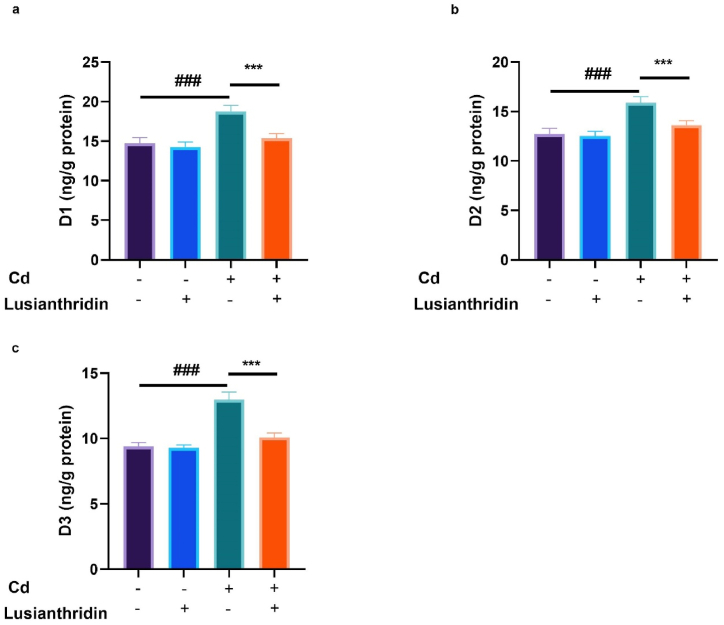
Showed the effect of Lusianthridin on the level of deiodinase against the cadmium induced thyroid cancer. **a:** D1, **b:** D2 and **c:** D3. Group III compared with the normal control and ^**###**^P < 0.001was consider as significant. Group IV compared with the Group III and ***P < 0.001was consider as significant.

Cd-induced thyroid cancer rats exhibited an improved level of D1 (Fig. 4a), D2 (Fig. 4b), and D3 (Fig. 4c), while Lusianthridin treatment significantly (P < 0.001) suppressed the level of deiodinase.

### 3.5 Hepatic parameters

**Fig. 5:**
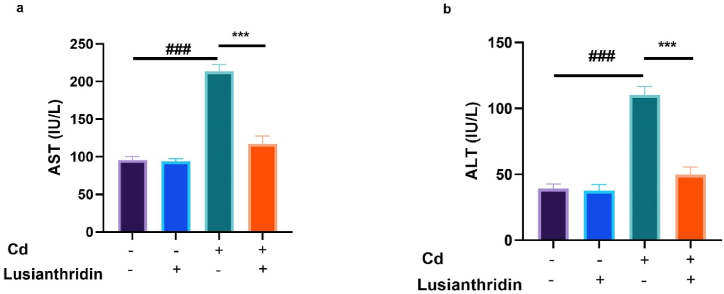
Showed the effect of Lusianthridin on the level of hepatic parameters against the cadmium induced thyroid cancer. **a:** AST and **b:** ALT. Group III compared with the normal control and ^**###**^P < 0.001was consider as significant. Group IV compared with the Group III and ***P < 0.001was consider as significant.

There is a complex relationship between thyroid cancer and liver function parameters. In some cases, thyroid cancer can lead to changes in liver enzymes and other hepatic parameters. Cd-induced thyroid cancer exhibited an elevated level of AST (Fig. 5a) and ALT (Fig. 5b). Lusianthridin remarkably (P < 0.001) suppressed the level of hepatic parameters.

### 3.6 Lipid parameters

**Fig. 6:**
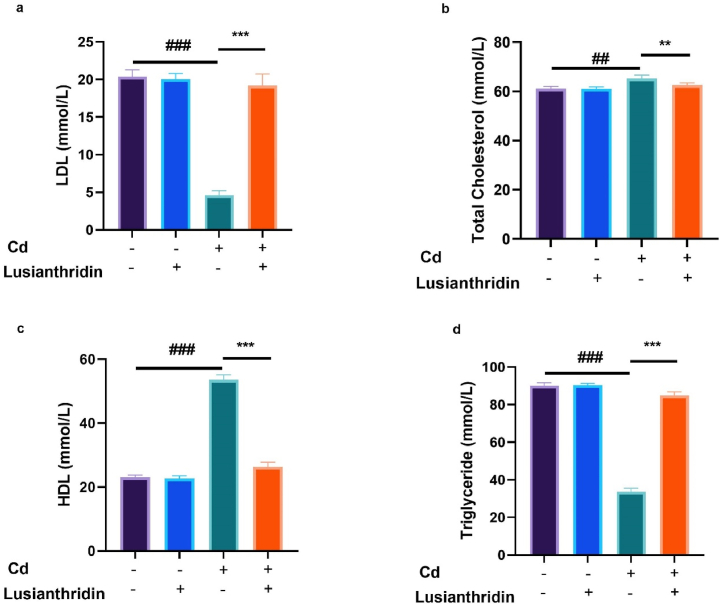
Showed the effect of Lusianthridin on the level of lipid parameters against the cadmium induced thyroid cancer. **a:** LDL, **b:** total cholesterol, **c:** HDL and **d:** triglyceride. Group III compared with the normal control and ^**###**^P < 0.001was consider as significant. Group IV compared with the Group III and ***P < 0.001was consider as significant.

Changes in lipid parameters can result from the impact of thyroid cancer on lipid metabolism. Thyroid hormones play an important role in regulating lipid metabolism, and abnormalities in thyroid function can affect lipid levels. The level of lipid parameters like LDL (Fig. 6a), total cholesterol (Fig. 6b), HDL (Fig. 6c) and triglycerides (Fig. 6d) were altered in Cd-induced thyroid cancer, and Lusianthridin considerably (P < 0.001) restored the level of lipid parameters.

### 3.7 Antioxidant marker

**Fig. 7:**
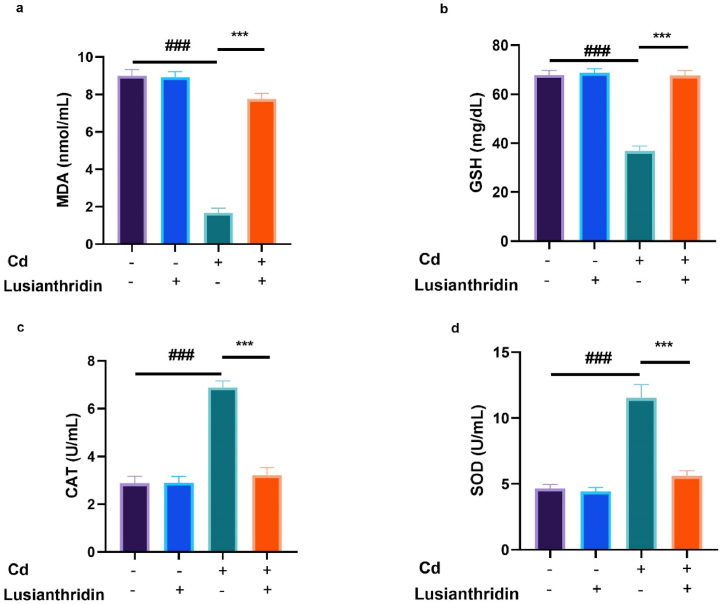
Showed the effect of Lusianthridin on the level of antioxidant parameters against the cadmium induced thyroid cancer. **a:** MDA, **b:** GSH, **c:** CAT and **d:** SOD. Group III compared with the normal control and ^**###**^P < 0.001was consider as significant. Group IV compared with the Group III and ***P < 0.001was consider as significant.

Antioxidants are substances that shield cells from damage caused by free radicals and other reactive oxygen species (ROS). These chemicals are essential in the care and prevention of several cancer forms, including thyroid cancer. The level of antioxidant markers such as MDA (Fig. 7a), GSH (Fig. 7b), CAT (Fig. 7c) and SOD (Fig. 7d) were altered in the Cd-induced group of rats, and Lusianthridin considerably (P < 0.001) restored the level of antioxidant parameters.

### 3.8 mRNA expression

**Fig. 8:**
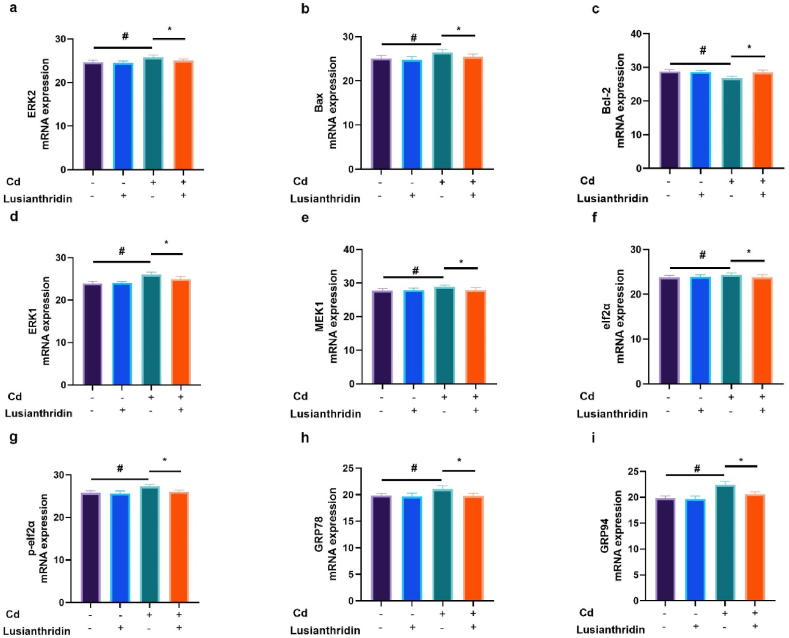
Showed the effect of Lusianthridin on the level of mRNA expression against the cadmium induced thyroid cancer. **a:** ERK4, **b:** Bax, **c:** Bcl-2**, d:** ERK1, **e:** MEK1, **f:** elf2α, **g:** p-elf2α, **h:** GRP78 and **i:** GRP94. Group III compared with the normal control and ^**###**^P < 0.001was consider as significant. Group IV compared with the Group III and ***P < 0.001was consider as significant.

Cd-induced thyroid cancer rats exhibited alterations in the level of mRNA expression viz., ERK4 (Fig. 8a), Bax (Fig. 8b), Bcl-2 (Fig. 8c)**,** ERK1 (Fig. 8d), MEK1 (Fig. 8e), elf2α (Fig. 8f), p-elf2α (Fig. 8g), GRP78 (Fig. 8h), GRP94 (Fig. 8i) and Lusianthridin treatment significantly (P < 0.001) restored the level of mRNA expression (Fig. 8).

## 4 Discussion

Heavy metal Cd is poisonous and can have a variety of negative impacts on biological systems. Cd toxicity is known to involve a number of different mechanisms, including interactions with metallothionein, oxidative damage, disruptions to calcium homeostasis, and alterations in gene expression [8,9,24]. One of the most well-characterized mechanisms of Cd toxicity is oxidative damage [25]. When cells are exposed to Cd, the generation of reactive oxygen species (ROS) can increase, and the antioxidant defense systems can become compromised, leading to oxidative damage to cellular macromolecules such as lipids, proteins, and DNA [18,20]. This can cause cellular dysfunction and tissue damage in various organs. Animal studies have demonstrated that Cd exposure can impair the function of organs, including the liver, kidneys, and lungs, resulting in morphological alterations in tissues and organelles such as mitochondria and lysosomes. These alterations can affect cellular metabolism, leading to cellular dysfunction and tissue damage [8,26]. Overall, there are several different processes involved in Cd toxicity, and the specific pathways can change based on the dose, duration, and exposure route. However, oxidative damage is a significant aspect of Cd toxicity and has been thoroughly investigated in relation to Cd-induced tissue dysfunction [8,27].

Thyroid hormones are essential for the formation, differentiation, growth, and maintenance of metabolic processes through thyroid hormone receptors [28,29]. Thyroid hormones play a crucial role in the growth and maturation of the nervous system. When these hormones are deficient at key stages of brain development, it can result in long-term cognitive and neurological problems. Additionally, thyroid hormones assist in controlling many other physiological processes, such as heart rate and body temperature, that are essential for preserving homeostasis in the body [30]. Thyroxine (T4) and triiodothyronine (T3), two thyroid hormones, are crucial for controlling metabolism and a variety of other bodily functions. T4 is the primary type of thyroid hormone circulating in the bloodstream, but T3 is the more active form and is more closely linked to the thyroid hormone receptors (TRs). The thyroid gland releases T4 into the blood, and deiodinase enzymes in various bodily tissues can transform it into T3 [31,32]. The conversion of T4 to T3 is an important mechanism for regulating the level of active thyroid hormone in different tissues. Thyroxine (T4) and triiodothyronine (T3) exert their effects by binding to specific nuclear receptors called thyroid hormone receptors (TRs), which are present in many different tissues throughout the body [33,34]. The thyroid hormone receptors (TRs) act as transcription factors, meaning that they help regulate the expression of many different genes by binding to specific DNA sequences in their promoter regions. As a result, the levels of many proteins and enzymes involved in metabolism, as well as other cellular processes like growth and differentiation, can alter [35,36]. In addition to regulating metabolic processes, thyroid hormones can also have an impact on the sex steroid endocrine system. Thyroid hormones can affect the production, transport, and metabolism of sex steroids such as estrogen and testosterone [37,38]. This may potentially have a role in the emergence and spread of a few hormone-dependent malignancies and can have a significant impact on reproductive function.

Biomarkers of antioxidant activity are important in thyroid cancer. Malondialdehyde (MDA) is a marker of lipid peroxidation, an oxidative stress that can harm cell membranes and promote the growth of cancer. Studies have shown that MDA levels are elevated in thyroid cancer patients compared to healthy controls, suggesting that oxidative stress may be involved in the pathogenesis of thyroid cancer [39]. The antioxidant enzyme SOD protects cells from oxidative stress by converting superoxide radicals into hydrogen peroxide. Studies have found that thyroid cancer patients had lower SOD levels, indicating that their antioxidant defense system may be weakened [40,41]. An antioxidant molecule called GSH (glutathione) guards cells against oxidative damage by scavenging free radicals. Studies have shown that thyroid cancer patients have lower GSH levels, which may indicate that their antioxidant defense mechanism is compromised [39]. An antioxidant enzyme called CAT breaks down hydrogen peroxide into water and oxygen. Studies have found that thyroid cancer patients had lower CAT levels, which may indicate that the patients' antioxidant defense mechanism is weak [41,42]. Overall, these biomarkers indicate that the pathogenesis of thyroid cancer may be influenced by oxidative stress and compromised antioxidant defense mechanisms. Antioxidant biomarker levels were altered in the Cd-induced thyroid cancer group rats, and Lusianthridin restored them to normal.

The thyroid hormones (THs) are essential for maintaining the body's energy homeostasis. The activity of the hypothalamus-pituitary-thyroid (HPT) axis, which controls the synthesis and secretion of THs, is one way that THs have an impact on the body [32]. By controlling the release of thyroid-stimulating hormone (TSH) from the pituitary gland and thyrotropin-releasing hormone (TRH) from the hypothalamus, thyroid hormones (THs) drive the hypothalamus-pituitary-thyroid (HPT) axis. TSH then stimulates the thyroid gland's production and release of THs. THs exert negative feedback on the HPT axis in this tightly regulated feedback loop to maintain consistent TH levels in the body. Disruption of the HPT axis can result in alterations in TH levels and disturbed energy homeostasis [37]. Disruption of the HPT axis may result in altered TH levels and increased TSH levels.

The thyroid gland, responsible for manufacturing thyroid hormones such as thyroxine (T4) and triiodothyronine (T3), can develop thyroid cancer. T3 and T4 play different roles in thyroid cancer depending on the disease's stage and type. The most common treatment for thyroid cancer is surgery to remove the thyroid gland, which may result in reduced thyroid hormone production. This can lead to hypothyroidism, which is typically treated with T4 replacement therapy. Due to its shorter half-life and more challenging management, T3 is not commonly used in thyroid hormone replacement treatment [43,44]. In some cases, thyroid cancer may lead to the overproduction of thyroid hormone, resulting in hyperthyroidism. This is observed in rare types of thyroid cancer, such as follicular thyroid carcinoma or papillary thyroid carcinoma, which produce excess amounts of T4. Treatment for these cases may involve thyroid hormone suppression therapy, wherein high doses of thyroid hormone (T4) are administered to suppress TSH production and reduce the growth of cancerous thyroid tissue [11]. The role of T3 and T4 in thyroid cancer is complex and depends on the stage and type of cancer. While T4 replacement medication is frequently used after thyroid gland surgery, hyperthyroidism caused by excessive T4 production may require suppression therapy to control cancer progression [[45], [46], [47]].

The biologically active forms of thyroid hormones are free triiodothyronine (FT3) and free thyroxine (FT4), and the hypothalamic-pituitary-thyroid (HPT) axis tightly controls the levels of these hormones. As the principal types of thyroid hormones generated and secreted by the thyroid gland, FT3 and FT4 play a comparable role to T3 and T4 in thyroid cancer [48]. The type and stage of cancer, as well as the treatments applied to manage the malignancy, can impact the levels of FT3 and FT4 in thyroid cancer. For instance, FT3 and FT4 levels may decline as thyroid cancer advances because the thyroid gland may become less effective at manufacturing thyroid hormones [37,38]. FT3 and FT4 levels may occasionally rise due to excessive thyroid hormone production by malignant thyroid tissue. Treatments for thyroid cancer, such as radioactive iodine therapy or thyroid hormone replacement therapy, may also impact FT3 and FT4 levels. Patients who have had their thyroid surgically removed may need lifelong T4 thyroid hormone replacement medication to keep their FT3 and FT4 levels within the normal range. Overall, the thyroid gland's activity plays a key part in FT3 and FT4's involvement in thyroid cancer, and various aspects of the disease and its therapy may impact the levels of these molecules [37,49]. Close monitoring of FT3 and FT4 levels is important for managing thyroid cancer and optimizing patient outcomes.

The results imply that exposure to Cd can cause thyroid follicular epithelial cells to undergo apoptosis (programmed cell death), which can contribute to the development of thyroid conditions, including thyroid cancer. The increased expression of ERK1, MEK1, *p*-eIf2, and GRP94, along with the decreased expression of Bcl-2, suggests that Cd-induced apoptosis is mediated by the activation of the ERK1/2 pathway and induction of endoplasmic reticulum stress [11,50,51]. However, the study also showed that fucoxanthin, a natural compound found in seaweed, can mitigate Cd-induced apoptosis by inhibiting the ERK1/2 pathway and preventing endoplasmic reticulum stress [11].

## 5 Conclusion

Lusianthridin demonstrated significant improvements in protein levels, T4, T3, and free iodine in urine, while concurrently suppressing the level of TSH. Furthermore, it exhibited noteworthy enhancements in the levels of FT3, FT4, and a reduction in the level of rT3. The compound also exerted a significant influence on deiodinase levels, particularly reducing D1, D2, and D3, and enhancing hepatic parameters like AST and ALT. Notably, Lusianthridin showcased remarkable alterations in lipid parameters, affecting LDL, total cholesterol, HDL, and triglycerides. The compound also demonstrated substantial effects on antioxidant parameters, influencing MDA, GSH, CAT, and SOD. The assessment of mRNA expression revealed significant changes in Bcl-2, Bax, MEK1, ERK1, ERK2, *p*-eIf2α, GRP78, eIf2α, and GRP94. Collectively, the results unequivocally indicate that Lusianthridin exhibits robust protective effects against thyroid cancer.

## Animal ethical

The study was conducted according to the guidelines of the Declaration of Helsinki and approved by the local ethics committee.

## Funding

N/A.

## Informed consent statement

Clear and Informed written consent was obtained from all donors involved in this study.

## Data availability statement

The data of the current study might be available on the request to the corresponding author.

## CRediT authorship contribution statement

**Teng Gao:** Writing – review & editing, Writing – original draft, Software, Investigation, Conceptualization. **Sijia Luo:** Writing – review & editing, Writing – original draft, Software, Resources, Project administration. **Hongguang Li:** Writing – review & editing, Writing – original draft, Validation, Supervision, Software, Project administration. **Zijie Su:** Writing – review & editing, Writing – original draft, Supervision, Project administration, Funding acquisition. **Qinghui Wen:** Writing – review & editing, Writing – original draft, Visualization, Validation, Supervision, Software.

## Declaration of competing interest

The authors declare none conflict of interest.
